# Supplementary material for: A Selective, Dual Emission β-Alanine Aminopeptidase Activated Fluorescent Probe for the Detection of Pseudomonas aeruginosa, Burkholderia cepacia, and Serratia marcescens
Source: Molecules. 2019 Sep 30;24(19):3550. doi: 10.3390/molecules24193550 (PMC6804094; doi:10.3390/molecules24193550)
Supplement: Supplementary file 1 [file molecules-24-03550-s001.pdf]

A Selective, Dual Emission β-Alanine Aminopeptidase Activated Fluorescent Probe for the Detection of *Pseudomonas aeruginosa, Burkholderia cepacia,* and *Serratia marcescens*

Supporting Information

Table S1 RGB and intensity values obtained by ImageJ RGB histogram on corresponding colonies of the multispot inoculated plates.

	Mean values (std.dev.)					NOTE
**Spot #**	**Intensity unweighted**	**Intensity weighted**	**Red**	**Green**	**Blue**	
1	45.63 (1.709)	40.86 (1.509)	23.62 (1.847)	44.092 (1.737)	69.202 (2.625)	Blueish reflection by lightsource
2	34.33 (1.987)	32.092 (1.566)	19.673 (1.780)	35.282 (1.7221)	47.869 (3.468)	
3	138.255 (36.082)	130.919 (37.747)	31.669 (16.495)	164.360 (47.473)	218.755 (45.440)	
4	151.333 (26.826)	144.505 (28.160)	47.961 (13.277)	177.266 (35.273)	228.713 (32.865)	
5	156.110 (40.411)	153.452 (42.669)	101.071 (39.620)	172.064 (45.647)	195.273 (36.821)	BAP-positive
6	109.362 (18.880)	102.484 (19.032)	23.619 (6.137)	128.374 (24.448)	176.067 (26.601)	
7	119.312 (15.203)	130.683 (18.840)	94.615 (18.595)	153.014 (21.713)	110.250 (8.701)	BAP-positive
8	79.438 (24.796)	76.952 (25.486)	18.539 (5.744)	98.011 (34.003)	121.658 (35.459)	
9	123.766 (41.493)	128.466 (45.154)	95.037 (41.792)	145.053 (49.191)	131.278 (33.934)	BAP-positive
10	91.170 (39.018)	86.429 (38.794)	21.261 (12.585)	108.486 (49.469)	143.830 (56.774)	Semi-inhibited
11	32.984 (1.191)	31.933 (1.152)	20.356 (1.247)	35.578 (1.121)	43.619 (1.563)	No growth
12	30.028 (1.284)	28.906 (1.178)	20.748 (1.161)	31.594 (1.333)	37.768 (2.120)	
13	31.578 (1.015)	30.509 (0.984)	20.064 (1.042)	33.954 (1.160)	41.578 (1.015)	
14	31.225 (1.118)	30.165 (1.098)	20.117 (1.251)	33.236 (1.245)	41 (1.725)	
15	42.250 (8.670)	40.667 (8.310)	17.128 (2.433)	48.734 (11.241)	60.835 (14.169)	Small semi-inhibited colonies
16	41.974 (8.122)	42.343 (8.987)	20.850 (2.944)	51.157 (12.409)	53.629 (10.051)	
17	49.269 (9.651)	49.293 (10.330)	18.243 (2.819)	61.424 (14.693)	68.312 (13.777)	
18	25.612 (1.008)	24.612 (1.008)	18.314 (1.025)	25.910 (1.183)	31.612 (1.008)	No growth
19	34.848 (7.228)	33.336 (6.597)	19.890 (2.190)	37.490 (8.137)	47.243 (12.171)	Very inhibited

20	23.964 (1.413)	23.536 (1.474)	18.195 (1.363)	25.267 (1.937)	28.655 (1.553)	No growth

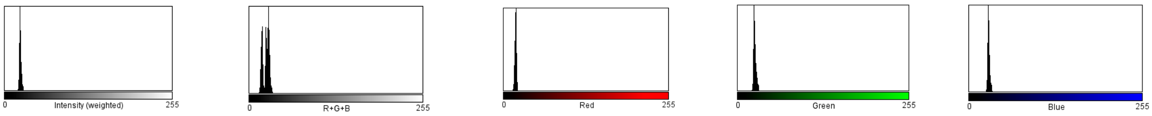

(**a**) (**b**) (**c**) (**d**) (**e**)

Figure S1 Histograms of (**a**) intensity, (**b**) RGB, (**c**) red, (**d**) green, (**e**) blue values for spot #20 as an example for typical **growth-inhibited** species

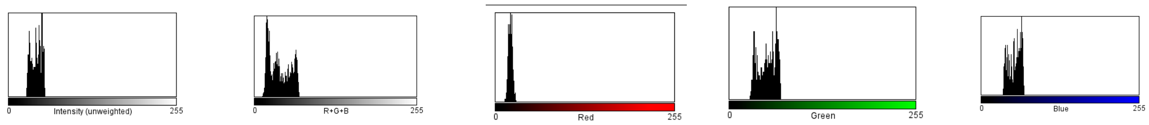

(**a**) (**b**) (**c**) (**d**) (**e**)

Figure S2 Histograms of (**a**) intensity, (**b**) RGB, (**c**) red, (**d**) green, (**e**) blue values for spot #16 as an example for **semi-inhibited BAP-negative** species

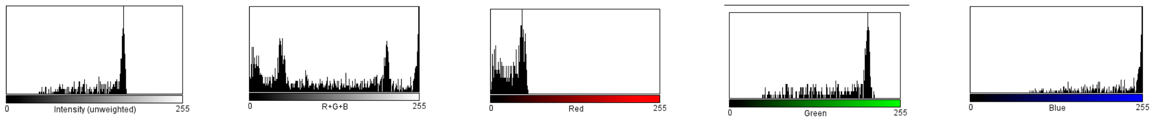

(**a**) (**b**) (**c**) (**d**) (**e**)

Figure S3 Histograms of (**a**) intensity, (**b**) RGB, (**c**) red, (**d**) green, (**e**) blue values for spot #3 as an example for **well-grown BAP-negative** species

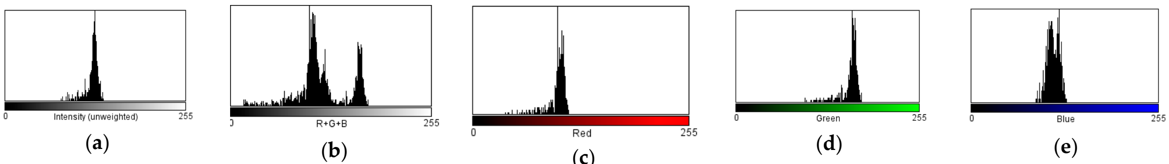

(**a**) (**b**) (**c**) (**d**) (**e**)

Figure S4 Histograms of (**a**) intensity, (**b**) RGB, (**c**) red, (**d**) green, (**e**) blue values for spot #7 ***P. aeruginosa*** **BAP-positive** strain

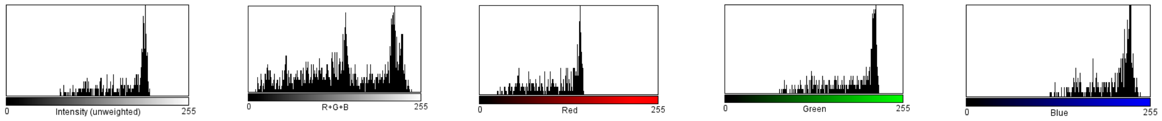

(**a**) (**b**) (**c**) (**d**) (**e**)

Figure S5 Histograms of (**a**) intensity, (**b**) RGB, (**c**) red, (**d**) green, (**e**) blue values for spot #5 ***S. marcescens*** **BAP-positive** strain

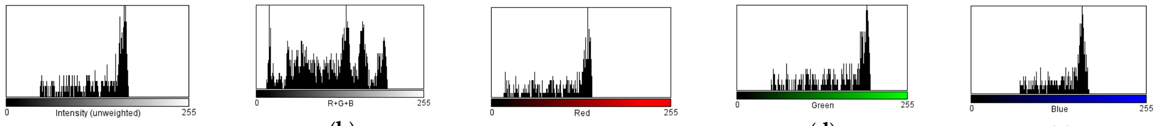

(**a**) (**b**) (**c**) (**d**) (**e**)

Figure S6 Histograms of (**a**) intensity, (**b**) RGB, (**c**) red, (**d**) green, (**e**) blue values for spot #9 ***B. cepacia*** **BAP-positive** strain

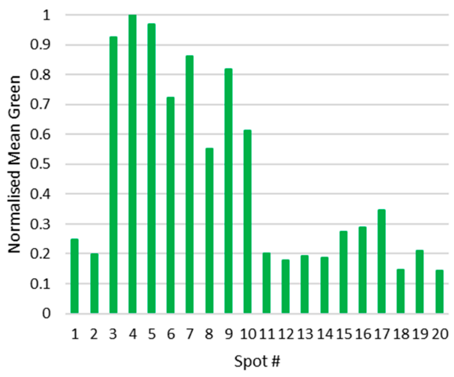

Growth/No growth indicator

(**a**)

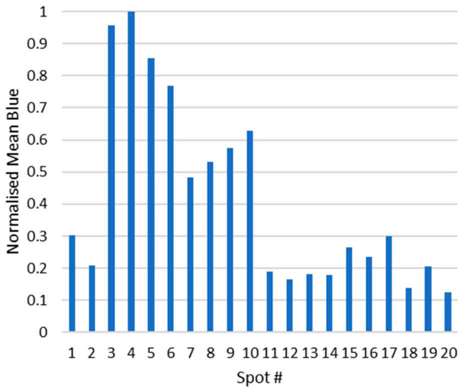

Growth/No growth indicator

(**b**)

Figure S7 Bar charts of ImageJ histogram values of the spot-inoculated colonies for each of the 20 strains of (**a**) green, and (**b**) blue component.

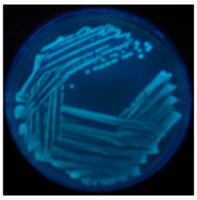

	Values
ΣIntensity	62.928
Red	13.772
Green	68.801
Blue	106.214
G/B	0.65
**R/B**	**0.13**
**R/I**	**0.22**

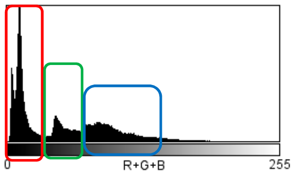

(a)

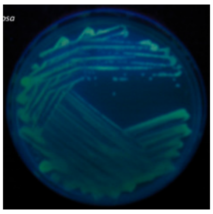

	Values
ΣIntensity	50.614
Red	22.786
Green	54.129
Blue	74.927
G/B	0.72
**R/B**	**0.30**
**R/I**	**0.45**

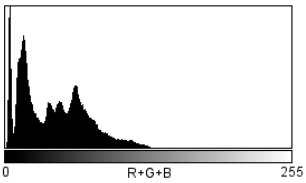

(b)

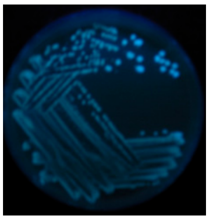

	Values
ΣIntensity	40.311
Red	13.343
Green	43.721
Blue	63.863
G/B	0.68
**R/B**	**0.21**
**R/I**	**0.33**

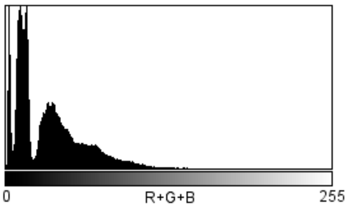

(c)

Figure S8 Streaked colonies; intensity, and respective R, G, B values and their ratios based on ImageJ image processing of the plates under a 365 nm light source, and RGB histograms of (**a**) *E. cloacae* (BAP-negative), (**b**) *P. aeruginosa* (BAP-positive), (**c**) mixed culture of *E. cloacae* and *P. aeruginosa*.

Characterisation

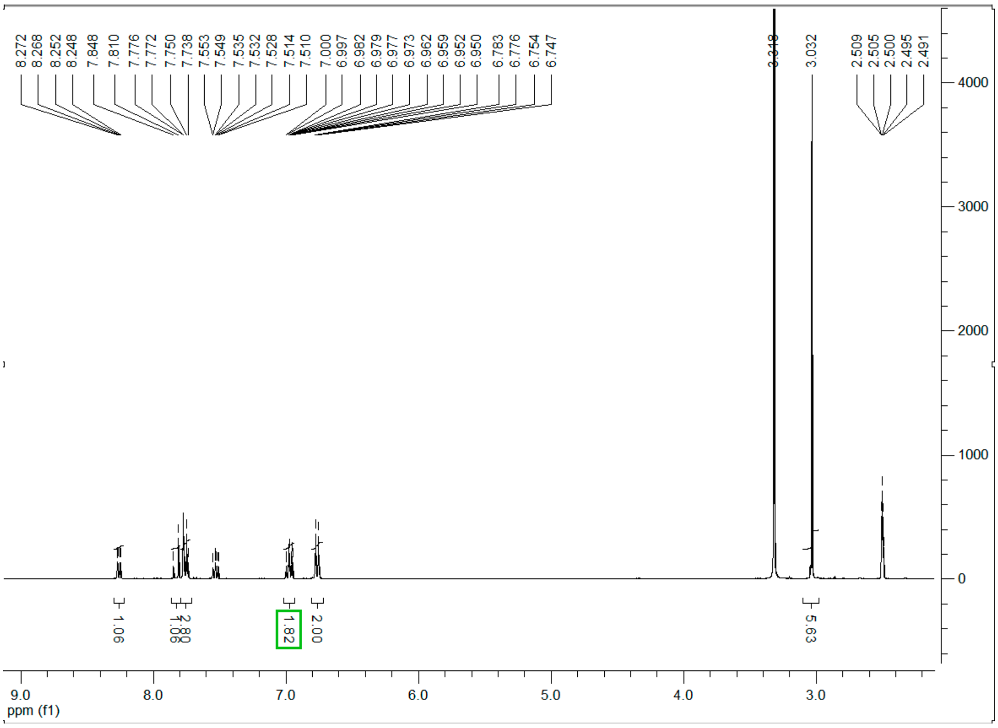

Figure S9 ^{1}H NMR of **10** in DMSO-d_6

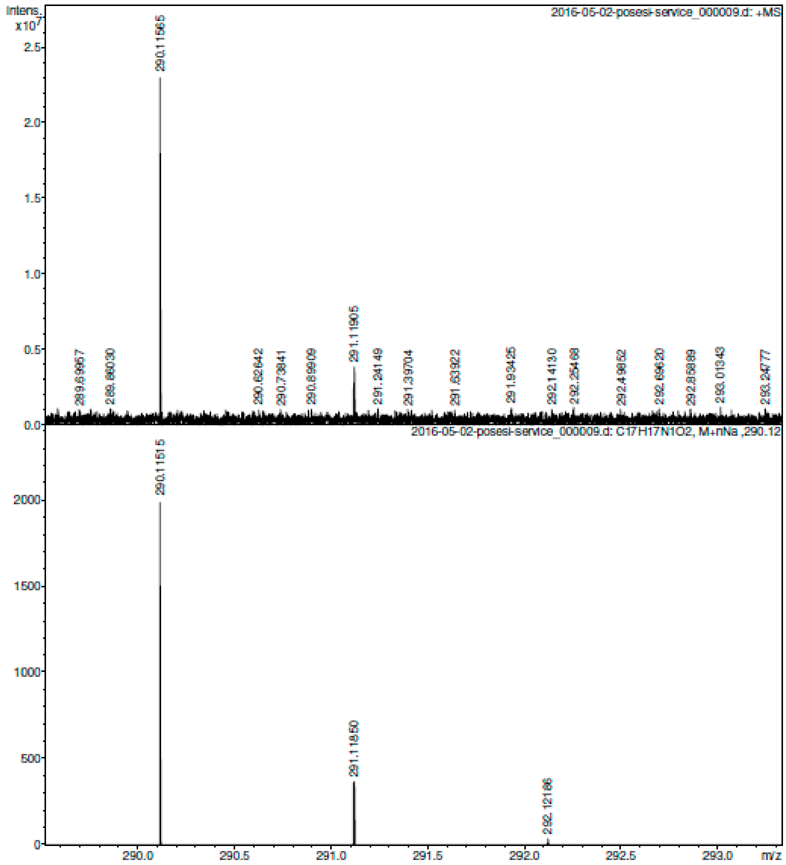

Figure S10 HRMS of **10**

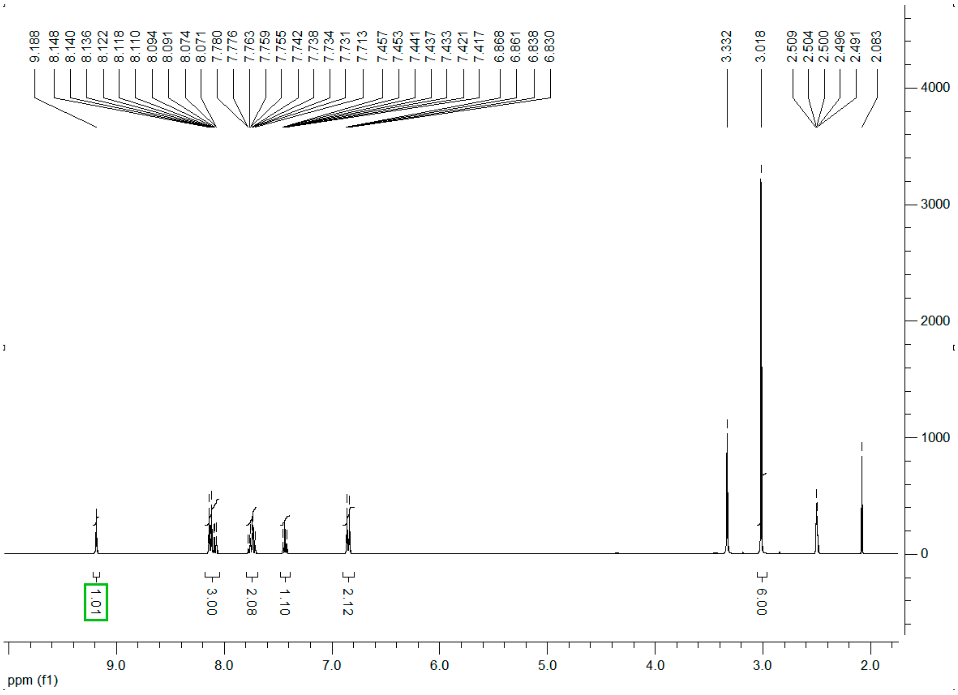

Figure S11 ^{1}H NMR of **7** in DMSO-d_6

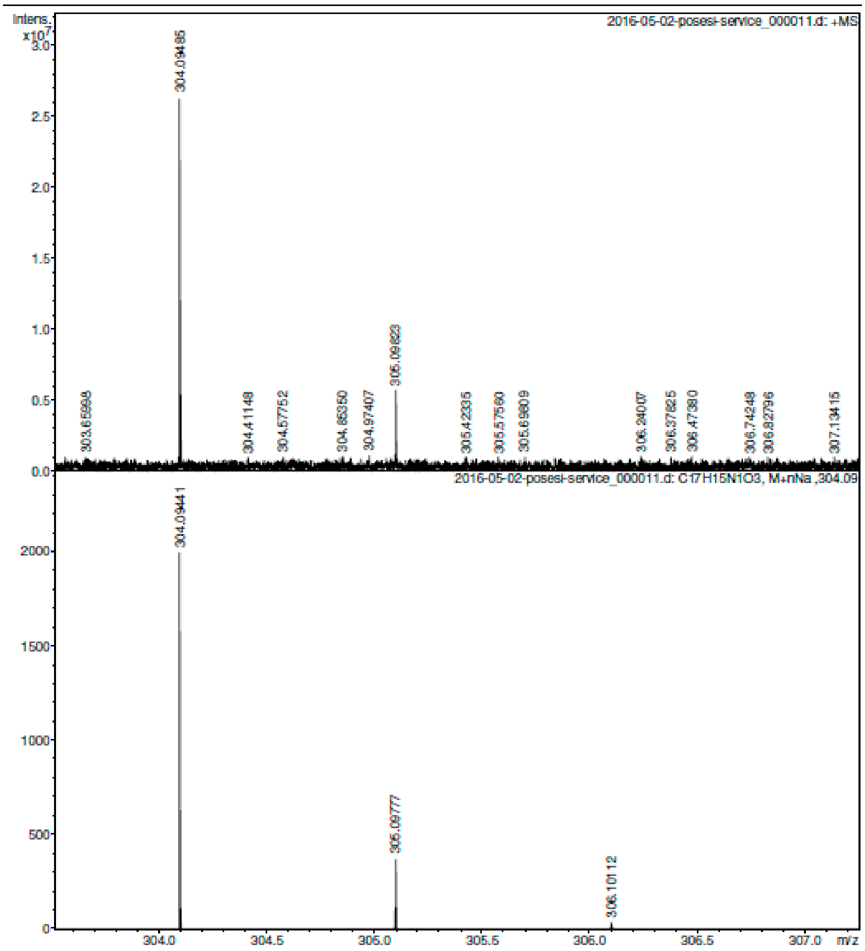

Figure S12 HRMS of **7**

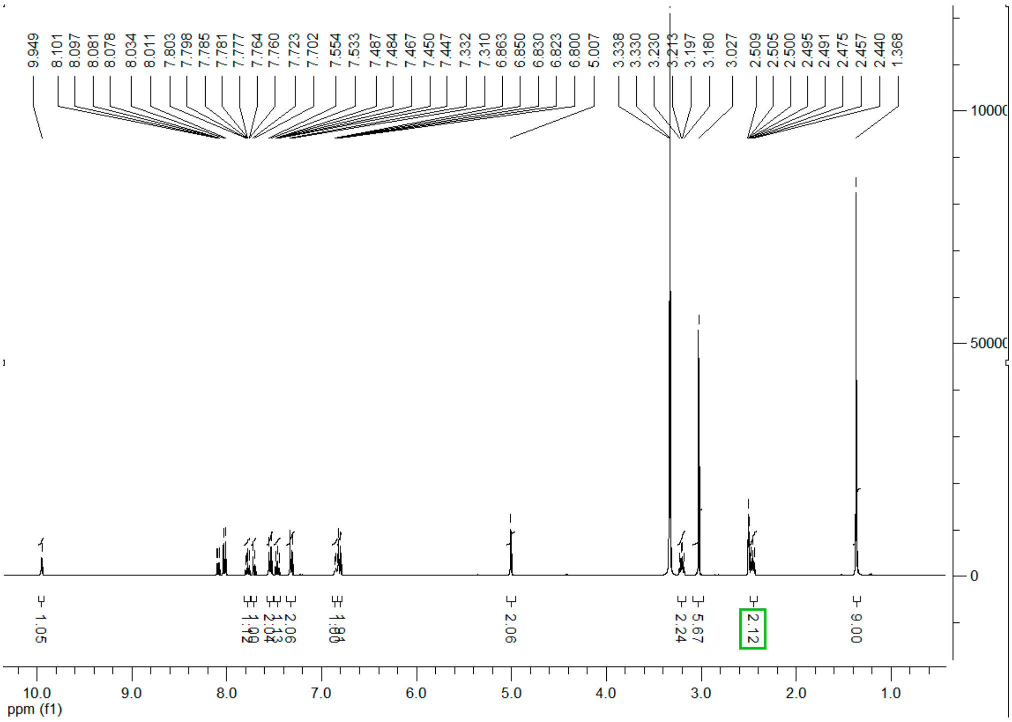

Figure S13 ^{1}H NMR of **12** in DMSO-d_6

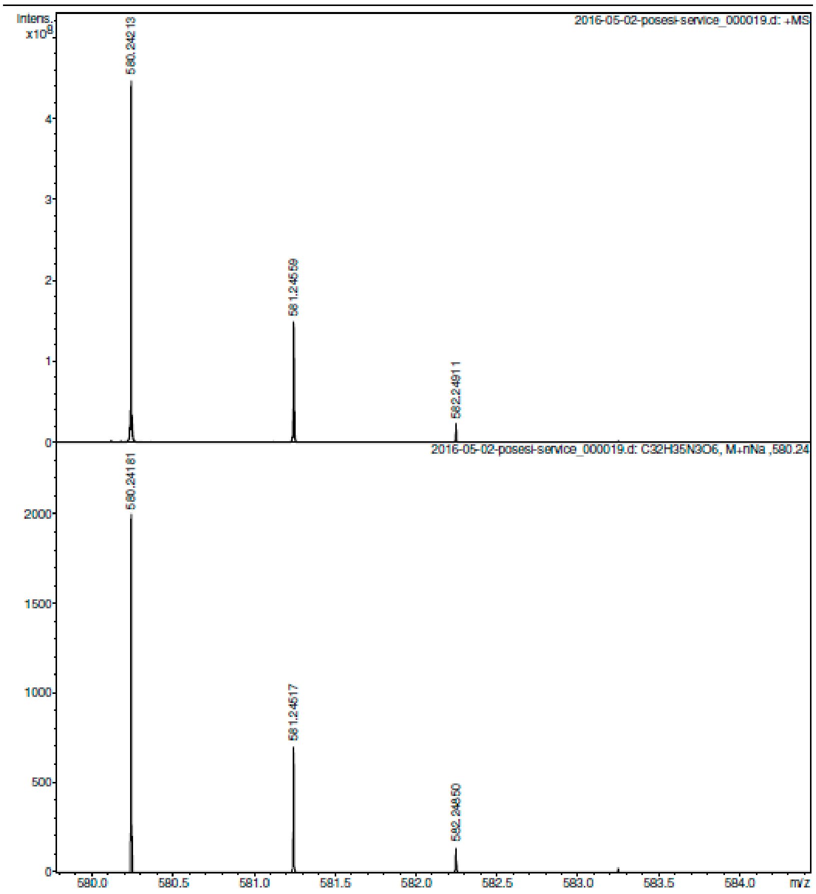

Figure S14 HRMS of **12**

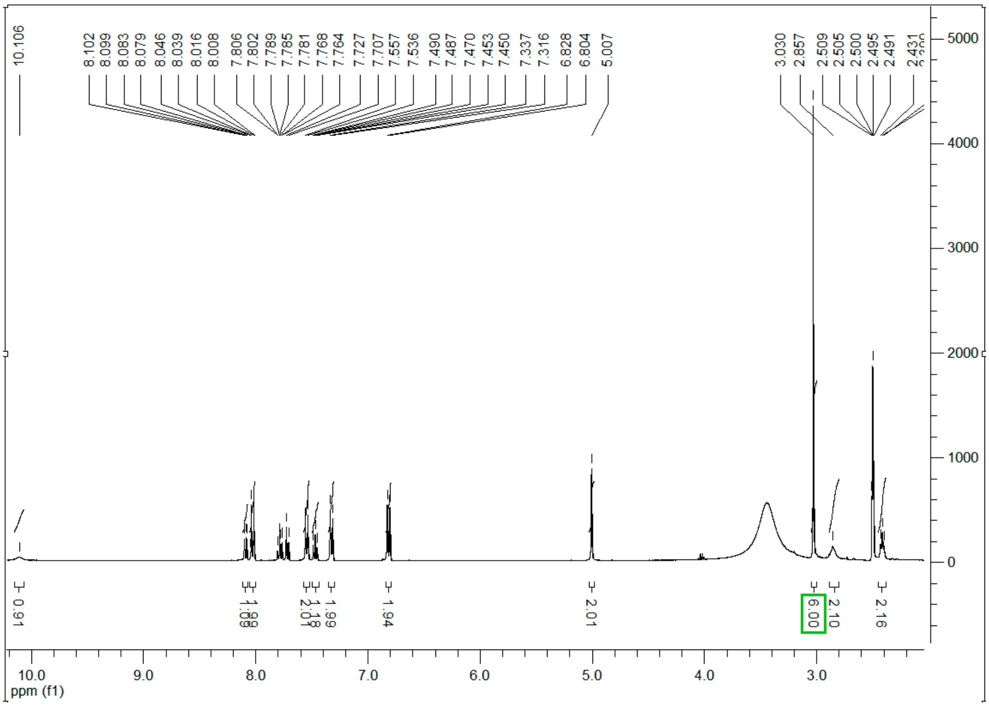

Figure S15 ^{1}H NMR of **9** in DMSO-d_6

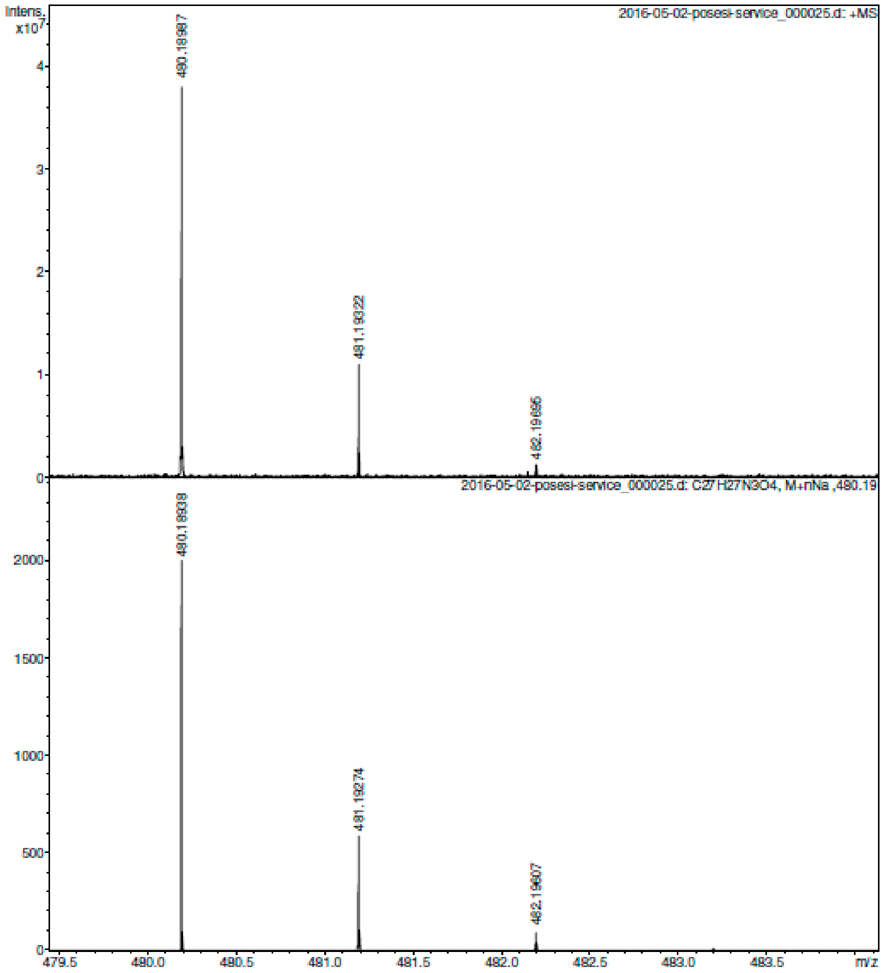

Figure S16 HRMS of **9**
